# Design and baseline characteristics of the Finerenone, in addition to standard of care, on the progression of kidney disease in patients with Non-Diabetic Chronic Kidney Disease (FIND-CKD) randomized trial

**DOI:** 10.1093/ndt/gfae132

**Published:** 2024-06-11

**Authors:** Hiddo J L Heerspink, Rajiv Agarwal, George L Bakris, David Z I Cherney, Carolyn S P Lam, Brendon L Neuen, Pantelis A Sarafidis, Katherine R Tuttle, Christoph Wanner, Meike D Brinker, Sara Dizayee, Peter Kolkhof, Patrick Schloemer, Paula Vesterinen, Vlado Perkovic, for the FIND-CKD investigators, Julio Bittar, Cesar Javier Zaidman, Natalia Cluigt, Miguel Hominal, Paola Aguerre, Fernando Halac, Elizabeth Gelersztein, Mariano Arriola, Rafael Maldonado, Mariano Chahin, David Packham, Darren Lee, Eugenia Pedagogos, Celine Foote, Sunil Badve, Carmel Hawley, Jenny Chen, Nicholas Gray, Marijn Speeckaert, Laura Labriola, Peter Doubel, Bart MAES, Kathleen Claes, Bernard Dubois, Irena Dimitrova, Tsvetelina Vutova, Stefan Ilchev, Svetla Stamova, Yordanka Ivanova, Albena Vasileva, Xiangmei Chen, Shuifu Tang, Xudong Xu, Bicheng Liu, Weiming He, Yani He, Fang Liu, Caili Wang, Lianhua Chen, Jianying Niu, Deguang Wang, Ping Luo, Yuou Xia, Gengru Jiang, Qun Luo, Fang Wang, Menghua Chen, Hongli Lin, Rui Yan, Yinan Li, Qinkai Chen, Junwu Dong, Fei Xiong, Haibo Long, Hong Cheng, Yuehong Li, Juan Du, Fanna Liu, Qingping Chen, Wanhong Lu, Chaosheng Chen, Jianqin Wang, Lei Liu, Min Yang, Gang Long, Yongjun Shi, Wenge Li, Xiangdong Yang, Aicheng Yang, Jianfei Li, Xiaoyan Meng, Martin Prazny, Lucie Hornova, Petr Bucek, Maria Majernikova, Jan Wirth, Jitka Rehorova, Mads Hornum, Jesper Bech, Morten Lindhardt, Ditte Hansen, Line Mortensen, Claus Juhl, Ioannis Boletis, Dorothea Papadopoulou, Evangelos Papachristou, Gerasimos Bamichas, Dimitrios Petras, Chariklia Gouva, Pantelis Sarafidis, Konstantinos Stylianou, Evangelia Ntounousi, Sydney Chi Wai Tang, Cheuk Chun Szeto, Samuel Ka Shun Fung, Sing Leung Lui, Laszlo Kovacs, Aniko Nemeth, Zsolt Zilahi, Tamas Szelestei, Robert Kirschner, Avinash Ignatius, Alan Almeida, Manisha Sahay, Subbiah Arunkumar, Dinesh Khullar, Rajendra Pandey, Sakthirajan Ramanathan, Noble Gracious, Siddharth Mavani, Nomy Levin-Iaina, Benaya Rozen-Zvi, Etty (Esther) Kruzel-Davila, Yosef Haviv, Sydney Ben Chetrit, Pazit Beckerman, Adi Leiba, Gil Chernin, Illia Beberashvili, Orit Kliuk-Ben Bassat, Yael Kenig, Evgeny Farber, Aneliya Parvanova Ilieva, Ciro Esposito, Roberto Minutolo, Gaetano La Manna, Gennaro Santorelli, Maria Cristina Gregorini, Gabriele Donati, Enrico Fiaccadori, Barbara Gidaro, Roberto Cimino, Giuseppe Grandaliano, Izaya Nakaya, Yoshitaka Maeda, Takayuki Toda, Hirokazu Okada, Morimasa Amemiya, Hitoshi Suzuki, Masanori Abe, Hiroshi Nishi, Yoshihiko Kanno, Seiji Ueda, Tetsuro Fujii, Jin Oshikawa, Masahiro Koizumi, Koichi Tamura, Masahiko Yazawa, Tamio Iwamoto, Tadashi Toyama, Kiyoki Kitagawa, Kohei Uchimura, Yuji Kamijo, Shinji Ako, Kanyu Miyamoto, Taro Misaki, Satoshi Suzuki, Hideaki Shimizu, Yoshiro Fujita, Minamo Ono, Atsushi Yamauchi, Hideki Fujii, Naohiko Fujii, Masaru Matsui, Kengo Kidokoro, Hidetoshi Kanai, Kosuke Masutani, Kiichiro Fujisaki, Masao Ishii, Megumi Nakamura, Mariko Toyoda, Yuichiro Makita, Li Yuan Lee, Chek Loong Loh, Suryati Yakob, Mohd Kamil Ahmad, Kai Quan Lee, Wan Ahmad Hafiz Wan Md Adnan, Muhamad Ali Sk Abdul Kader, Nuzaimin Hadafi Ahmad, Subasni Govindan, Mohamad Zaimi Abdul Wahab, Sadanah Aqashiah Datuk Mazlan, Sergio Irizar Santana, Alfredo Chew Wong, Sandro Avila Pardo, Edmundo Bayram, Rita Birne, Fernando Teixeira e Costa, Joana Silva Costa, Ana Rita Alves, Tiago Pereira, Tatyana Rodionova, Natalia Antropenko, Tatyana Abissova, Elena Zhdanova, Andrey Ezhov, Sufi Muhummad Suhail, Allen Liu, Jimmy Teo, See Cheng Yeo, Ngiap Chuan Tan, SungGyun Kim, Kang Wook Lee, Seok Joon Shin, Byoung-Geun Han, Jangwook Lee, Sang Youb Han, Hye Ryoun Jang, Jung Pyo Lee, Jung Tak Park, Young Sun Kang, So Young Lee, Yong Chul Kim, Sang Ho Lee, Hayne Park, Ji Eun Oh, Yeong Hoon Kim, Bum Soon Choi, Jose Julian Segura de la Morena, Julio Hernandez Jaras, Francisco Martínez Debén, Hanane Bouarich, Pau Llacer Iborra, María Soler Romero, Jose Gorriz Teruel, Cristina Castro, Josep Cruzado Garrit, Clara Barrios, Yen-Ling Chiu, Hsi-Hsien Chen, Cheng-Chieh Hung, Shuei-Liong Lin, Chien-Te Lee, Ming-Ju Wu, Ping-Fang Chiu, Chiz-Tzung Chang, Hui-Teng Cheng, Kieran McCafferty, Siân Griffin, Priscilla Smith, Tim Doulton, Thomas Pickett, Arif Khwaja, Radica Alicic, Sreedhara Alla, Sanjiv Anand, Mohamed Atta, Ahmed Awad, Shweta Bansal, Anna Burgner, Alex Chang, Cynthia Christiano, Aditi Gupta, German Hernandez, Aamir Jamal, Eric Kirk, Nelson Kopyt, Wayne Kotzker, Ramon Mendez, Jill Meyer, Ahmadshah Mirkhel, George Newman, Sagar Panse, Pablo Pergola, Mahboob Rahman, Anjay Rastogi, Mark Smith, Jeffrey Turner, Guillermo Umpierrez, Nam Vo, Darren Schmidt, Adam Frome, George Nakhoul, Ronald Ralph, Jonathan Tolins, Jessica Kendrick, Michael Quadrini, Sadaf Elahi, Sergio Trevino Manllo, Wen-Yuan Chiang, Jany Moussa, Tina Thethi

**Affiliations:** Department of Clinical Pharmacy and Pharmacology, University of Groningen, Groningen, The Netherlands; Division of Nephrology, Department of Medicine, Indiana University School of Medicine and RL Roudebush VA Medical Center, Indianapolis, IN, USA; Department of Medicine, AHA Comprehensive Hypertension Center, University of Chicago Medicine, Chicago, IL, USA; Department of Medicine, Division of Nephrology, University Health Network, Toronto, ON, Canada; National Heart Centre Singapore, Singapore, Singapore; Duke-National University of Singapore Medical School, Singapore, Singapore; Renal and Metabolic Division, George Institute for Global Health, University of New South Wales, Sydney, New South Wales, Australia; Department of Renal Medicine, Royal North Shore Hospital, Sydney, New South Wales, Australia; 1st Department of Nephrology, Aristotle University of Thessaloniki, Hippokration Hospital, Thessaloniki, Greece; Providence Inland Northwest Health, Spokane, WA, USA; Nephrology Division and Kidney Research Institute, University of Washington School of Medicine, Seattle, WA, USA; Department of Clinical Research and Epidemiology, Comprehensive Heart Failure Center, University of Würzburg, Würzburg, Germany; Cardiology and Nephrology Clinical Development, Bayer AG, Wuppertal, Germany; Regulatory Strategy Cardiology and Nephrology, Bayer AG, Wuppertal, Germany; Cardiovascular Precision Medicines, Research and Development, Bayer AG, Wuppertal, Germany; Clinical Statistics & Analytics, Research and Development, Bayer AG, Berlin, Germany; Medical and Scientific Affairs, Bayer Pharmaceuticals, Espoo, Finland; Renal and Metabolic Division, George Institute for Global Health, University of New South Wales, Sydney, New South Wales, Australia

**Keywords:** clinical trial, eGFR slope, finerenone, non-diabetic chronic kidney disease, immunoglobulin A nephropathy

## Abstract

**Background:**

Finerenone, a non-steroidal mineralocorticoid receptor antagonist, improved kidney and cardiovascular outcomes in patients with chronic kidney disease (CKD) and type 2 diabetes in two phase 3 outcome trials. The Finerenone, in addition to standard of care, on the progression of kidney disease in patients with Non-Diabetic Chronic Kidney Disease (FIND-CKD) study investigates the effect of finerenone in adults with CKD without diabetes.

**Methods:**

FIND-CKD (NCT05047263 and EU CT 2023-506897-11-00) is a randomized, double-blind, placebo-controlled phase 3 trial in patients with CKD of non-diabetic aetiology. Adults with a urinary albumin:creatinine ratio (UACR) ≥200–≤3500 mg/g and an estimated glomerular filtration rate (eGFR) ≥25–<90 ml/min/1.73 m^2^ receiving a maximum tolerated dose of a renin–angiotensin system inhibitor were randomized 1:1 to once-daily placebo or finerenone 10 or 20 mg depending on eGFR >60 or <60 ml/min/1.73 m^2^. The primary efficacy outcome is total eGFR slope, defined as the mean annual rate of change in eGFR from baseline to month 32. Secondary efficacy outcomes include a combined cardiorenal composite outcome comprising time to kidney failure, sustained ≥57% decrease in eGFR, hospitalization for heart failure or cardiovascular death, as well as separate kidney and cardiovascular composite outcomes. Adverse events are recorded to assess tolerability and safety.

**Results:**

Across 24 countries, 3231 patients were screened and 1584 were randomized to study treatment. The most common causes of CKD were chronic glomerulonephritis (57.0%) and hypertensive/ischaemic nephropathy (29.0%). Immunoglobulin A nephropathy was the most common glomerulonephritis (26.3% of the total population). At baseline, mean eGFR and median UACR were 46.7 ml/min/1.73 m^2^ and 818.9 mg/g, respectively. Diuretics were used by 282 participants (17.8%), statins by 851 (53.7%) and calcium channel blockers by 794 (50.1%). Sodium–glucose co-transporter 2 (SGLT2) inhibitors were used in 16.9% of patients; these individuals had a similar mean eGFR (45.6 versus 46.8 ml/min/1.73 m^2^) and a slightly higher median UACR (871.9 versus 808.3 mg/g) compared with those not using SGLT2 inhibitors at baseline.

**Conclusions:**

FIND-CKD is the first phase 3 trial of finerenone in patients with CKD of non-diabetic aetiology.

## Graphical Abstract

**Graphical Abstract:**
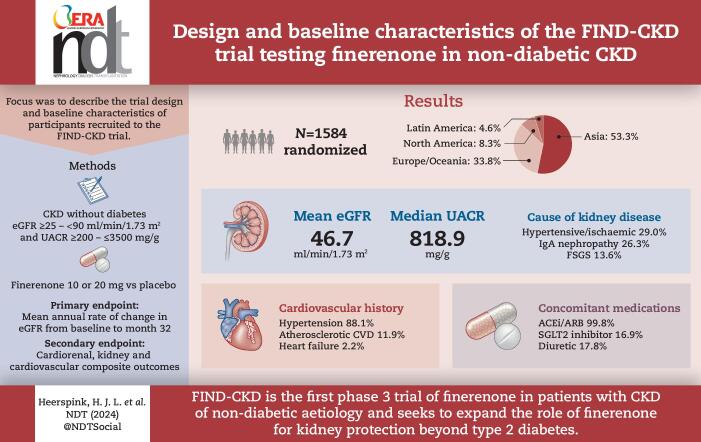

KEY LEARNING POINTS **What was known:**

- Finerenone reduced the risk of kidney failure and improved cardiovascular outcomes in patients with chronic kidney disease (CKD) associated with type 2 diabetes (T2D) in the phase 3 FIDELIO-DKD and FIGARO-DKD outcome trials.
- Finerenone acts by targeting pathways of pathophysiological sodium retention, inflammation and fibrosis mediated by mineralocorticoid receptor overactivation in the vasculature, heart and kidney.
- Based on its properties, finerenone may also be beneficial in improving kidney and cardiovascular outcomes in patients with CKD without diabetes. This hypothesis is being tested in the ongoing FIND-CKD trial.

**This study adds:**

- The FIND-CKD trial is the first phase 3 trial studying the efficacy of finerenone in patients with CKD of non-diabetic aetiology, including hypertension and chronic glomerulonephritis (e.g. immunoglobulin A nephropathy and focal segmental glomerulosclerosis), who are at risk of progression.
- The primary endpoint in FIND-CKD is the estimated glomerular filtration rate slope at 32 months.
- The utilization of a hybrid decentralized clinical trial model is an added novelty that offers study participants and sites increased flexibility in trial conduct. This model integrates telemedicine technology into certain parts of the clinical research process and supports management of research activities, including remote data collection.

**Potential impact:**

- The FIND-CKD trial will determine the efficacy of finerenone for slowing kidney disease progression in patients with CKD without diabetes, providing a potential expanded role for finerenone for the treatment of CKD beyond T2D.

## INTRODUCTION

Chronic kidney disease (CKD) affected ≈840 million people around the world in 2017 [1]. CKD due to diabetes is the most common cause of kidney failure, but a substantial proportion of

patients (50–80%) have CKD of other aetiologies [2, 3]. While the pathogenesis underlying the causes of these different CKD aetiologies are different, the pathophysiological events contributing to the progression of most of the common non-diabetic CKD aetiologies are, to a certain extent, similar and involve glomerular hyperfiltration, proteinuria, interstitial inflammation and, ultimately, fibrosis [3].

The current pharmacological strategy employed to reduce the rate of glomerular filtration rate (GFR) decline in patients with CKD involves inhibition of the renin–angiotensin system (RAS) with angiotensin-converting enzyme inhibitors (ACEIs) and angiotensin receptor blockers (ARBs) [4]. The existing evidence for ACEIs and ARBs is mainly derived from global clinical trials with ARBs in patients with CKD and diabetes [5, 6]. There are no international clinical trials, involving a globally diverse population, on the efficacy and safety of these therapies in patients with CKD without diabetes. The advent of sodium–glucose co-transporter 2 (SGLT2) inhibitors on top of RAS inhibition has further slowed the progression of kidney function decline in patients with CKD with and without diabetes [7, 8]. While patient prognosis has markedly improved in recent years, the risks of kidney failure and cardiovascular complications remain very high, especially for certain subgroups [9]. For example, in the EMPA-KIDNEY trial (NCT03594110), the rate of chronic estimated GFR (eGFR) decline among patients with CKD and severe albuminuria (>300 mg/g) randomized to empagliflozin was −2.35 ml/min/1.73 m^2^/year [7], more than 3-fold higher compared with the average rate of decline associated with ageing [10].

Finerenone is a potent, selective, non-steroidal mineralocorticoid receptor antagonist (MRA) that targets haemodynamic (sodium retention, blood pressure and albuminuria) and non-haemodynamic (inflammatory and fibrotic) pathways mediated by mineralocorticoid receptor overactivation in the vasculature, heart and kidney [11, 12]. In phase 3 clinical trials [FIDELIO-DKD (NCT02540993) and FIGARO-DKD (NCT02545049)] enrolling patients with CKD and type 2 diabetes (T2D), finerenone reduced the risks of kidney and cardiovascular events [13, 14]. In the FIDELIO-DKD trial, finerenone reduced the risk of an established composite kidney endpoint (comprising a 57% eGFR decline from baseline, kidney failure or death from kidney causes) by 24% and an eGFR decline by 0.65 ml/min/1.73 m^2^/year compared with placebo [15]. This difference in eGFR slope between treatment arms was more pronounced in patients with an elevated urinary albumin:creatinine ratio (UACR ≥500 mg/g).

The Finerenone Non-Diabetic Chronic Kidney Disease (FIND-CKD) trial is a phase 3 randomized controlled trial of finerenone in patients with CKD of non-diabetic aetiology. The FIND-CKD trial is designed to test the hypothesis that finerenone is superior to placebo in reducing eGFR decline in patients with CKD without diabetes already receiving an optimized dose of an ACEI or ARB as standard-of-care kidney protective therapy. This trial will determine the potential expanded role of finerenone for the treatment of CKD of non-diabetic aetiology.

## MATERIALS AND METHODS

### Study design

FIND-CKD is a multicentre, randomized, double-blind, placebo-controlled, parallel-group phase 3 study in patients with non-diabetic CKD of different disease aetiologies who are at risk of progressive eGFR decline. The primary objective is to demonstrate whether the addition of finerenone to the standard of care (optimized RAS blockade) is superior to placebo in slowing the progression of kidney disease in patients with CKD without diabetes. The study is being conducted in compliance with the principles of the Declaration of Helsinki and in accordance with the protocol and the International Conference on Harmonization Guidelines for Good Clinical Practice. The study protocol, any protocol amendments and informed consent forms are subject to approval following review by health authorities, independent review boards and independent ethics committees according to country-specific requirements. All study patients provided written informed consent prior to study enrolment. The study is registered with ClinicalTrials.gov (www.clinicaltrials.gov; NCT05047263) and the European Clinical Trial Register (EU CTR 2023-506897-11-00). It is estimated that the study will be completed in February 2026.

### Study patients

**Figure 1:**
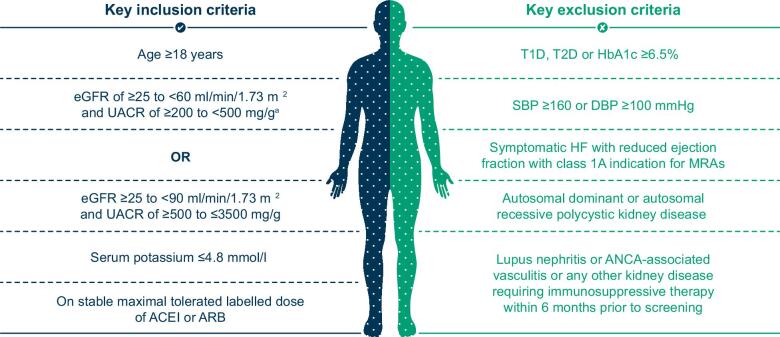
Key eligibility criteria. ^a^To ensure a pre-specified ratio for a population at risk of progressive renal function decline, the number of participants with an eGFR ≥25–<60 ml/min/1.73 m^2^ and UACR ≥200–<500 mg/g is planned to be capped at ≈10% of the total population. DBP: diastolic blood pressure; HbA1c: glycated haemoglobin; HF: heart failure; SBP: systolic blood pressure; T1D: type 1 diabetes.

Patients ≥18 years of age with a clinical diagnosis of CKD were eligible for the study if they had either an eGFR ≥25–<60 ml/min/1.73 m^2^ and a UACR ≥200–<500 mg/g or an eGFR ≥25–<90 ml/min/1.73 m^2^ and a UACR ≥500–≤3500 mg/g. For inclusion, patients had to be on a stable and maximum tolerated dose of an ACEI or ARB for at least 4 weeks and have a serum potassium value ≤4.8 mmol/l. Full eligibility criteria are outlined in Supplementary data Table S1 and Fig. 1. Patients with an established diagnosis of type 1 diabetes, T2D or glycated haemoglobin ≥6.5% were not eligible for enrolment. Other exclusion criteria include systolic blood pressure ≥160 mmHg or diastolic blood pressure ≥100 mmHg, symptomatic heart failure with reduced ejection fraction with class 1A indication for steroidal MRAs, autosomal dominant or autosomal recessive polycystic kidney disease, lupus nephritis or anti-neutrophilic cytoplasmic autoantibody (ANCA)-associated vasculitis or any other kidney disease requiring immunosuppressive therapy within 6 months prior to enrolment.

A screening visit was scheduled for each patient 3–14 days before randomization, with an optional prescreening visit to identify potential patients prior to main study consent.

### Randomization and study treatment

**Figure 2:**
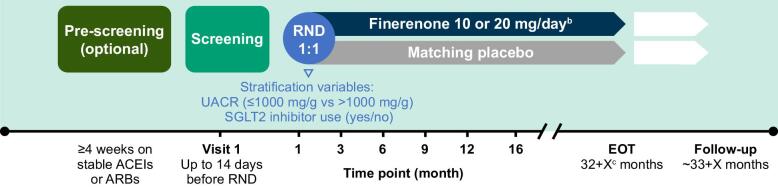
Study design of FIND-CKD^a^. ^a^Study duration and the number of study visits will depend on the time of enrolment of the patient. ^b^Starting dose of finerenone is based on the patient's eGFR level at the screening visit. Finerenone will be up- or down-titrated based on potassium and eGFR levels. ^c^Participants will stay in the study until the last randomized participant has reached 32 months of treatment. EOT: end of treatment; RND: randomization.

Patients were randomized 1:1 to either finerenone (10 or 20 mg) or placebo (sham 10 or 20 mg) once daily (OD) in a blinded fashion on top of their stable standard-of-care therapy (including stable and maximum tolerated dose of ACEI or ARB therapy; Fig. 2). Randomization was stratified by screening UACR (≤1000 mg/g versus >1000 mg/g) and baseline SGLT2 inhibitor use (yes/no). The starting dose depends on the participant's eGFR level: a lower dose of 10 mg OD if eGFR is ≥25–<60 ml/min/1.73 m^2^ or the higher (target) dose of 20 mg OD if eGFR is ≥60 ml/min/1.73 m^2^. Finerenone will be up- or down-titrated based on potassium and eGFR levels.

### Study follow-up

Visits were scheduled at screening, baseline (day 1), month 1 and month 3, then every 3 months up to month 12 (Fig. 2). Thereafter, 4-month visits were planned until the end of the study. The minimum planned treatment duration of each patient is 32 months and the maximum is ≈49 months for the first randomized participant. An additional visit is scheduled 4 weeks after study drug discontinuation to assess off-drug effects.

### Efficacy and safety assessments

Demographic characteristics and medical history were recorded at screening. FIND-CKD utilizes a 3-month visit schedule during the first year, followed by a 4-month visit schedule thereafter, with the aim of comprehensively characterizing the initial eGFR slope with finerenone and to adequately determine the point of change from acute to chronic eGFR slope. The primary efficacy variable, eGFR, is assessed at all study visits, including the follow-up visit. eGFR is assessed by a central laboratory. The formula used for calculating eGFR (in ml/min/1.73 m^2^) is the 2009 Chronic Kidney Disease Epidemiology Collaboration (CKD-EPI) equation based on creatinine, and including age, sex and race [16]. In addition, prespecified sensitivity analyses for eGFR will be performed using the 2021 equations [17]. Cystatin C measurements are collected at all time points of creatinine measurements in a central laboratory for this purpose.

UACR is measured at screening; day 1; months 1, 3, 6, 12 and 24; the end of the planned treatment period and the follow-up visit to assess effects after cessation of treatment. UACR is evaluated using first morning void urine samples collected at the patient's home on three consecutive days before the screening visit, on three consecutive days before the day 1 visit and on two consecutive days before the other scheduled visits. UACR is assessed by a central laboratory. In addition, urinary protein:creatinine ratio is assessed at the same time points. Blood pressure is assessed at all scheduled visits. Health status is assessed using the EuroQol five dimension, five level questionnaire (EQ-5D-5L) [18], completed on day 1, then every 12 months until the end of the planned treatment period.

Safety is assessed at all scheduled visits and includes assessment of vital signs and adverse events. A 12-lead electrocardiogram is performed on day 1 and at the end of the planned treatment period. Hyperkalaemia is an adverse event of special interest in this study. Serum potassium and hyperkalaemia are characterized through monitoring the change from baseline in serum potassium and identification of the number of patients with serum potassium levels >5.5 mmol/l and >6.0 mmol/l.

### Statistical analysis

#### Sample size determination

A total sample size of 1500 patients (750 per treatment arm) was calculated to have >90% power to demonstrate the superiority of finerenone to placebo with respect to total eGFR slope from baseline on day 1 to month 32 at a two-sided significance level of 5%. The >90% power calculation assumes a 0.7 ml/min/1.73 m^2^/year total eGFR slope difference at month 32 of treatment. The assumption and initial parameter estimate of total eGFR slope are based on findings demonstrating that a yearly eGFR slope reduction of 0.5–1.0 ml/min/1.73 m^2^ is associated with a hazard ratio (HR) of ≈0.7 for clinical outcomes in moderate–large randomized trials [19]. Screening ≈3160 patients was planned to achieve 1580 patients randomly assigned to the study intervention. This sample size would allow for an ≈5% overall discontinuation rate in the study (≈40 patients in each treatment arm).

#### Primary outcome

The primary efficacy outcome is total eGFR slope, defined as the mean annual rate of change in eGFR from baseline to month 32. For analysis of the total slope, the serial change in eGFR will be modelled using a two-slope linear spline mixed effects model in which a fixed change point will define acute and chronic eGFR slope at month 3 [20]. The acute and chronic phases are differentiated because finerenone showed acute effects on eGFR that differed from its long-term effects on disease progression in FIDELIO-DKD [13] and FIGARO-DKD [14]. In addition to fixed effects for treatment (finerenone versus placebo), time (continuous), a treatment-by-time interaction and baseline eGFR (continuous), the model will adjust for stratification factors used at randomization [i.e. baseline SGLT2 inhibitor use (yes/no) and screening visit UACR (≤1000 mg/g versus >1000 mg/g)]. A random effect for the intercept, acute eGFR slope (baseline to month 3) and chronic eGFR slope (month 3 to the planned end of the treatment period) will be included to account for variations in progression rates of patients over time. The total eGFR slope difference of finerenone and placebo will be estimated at month 32, considering the anticipated data availability and duration of treatment. The study will be considered successful if the *P*-value is statistically significant (*P* < .05). The primary analysis of the primary efficacy variable will be conducted using the full analysis set, comprising all randomized patients. In addition, chronic eGFR slope (from month 3 to the end of treatment) and change in eGFR from baseline to 4 weeks after the end of treatment will further supplement the primary endpoint analysis with the aim of characterizing the long-term effects of finerenone.

#### Secondary outcomes

The secondary efficacy outcome is a combined cardiorenal endpoint comprising the onset of kidney failure (end-stage kidney disease or sustained decrease in eGFR <15 ml/min/1.73 m^2^), a sustained ≥57% decrease in eGFR from baseline, hospitalization for heart failure or death from cardiovascular-related causes. Additional secondary efficacy outcomes include a kidney composite outcome comprising the onset of kidney failure or sustained ≥57% decrease in eGFR from baseline and a cardiovascular composite endpoint comprising hospitalization for heart failure or death from cardiovascular-related causes. The null hypotheses of the secondary endpoints will be tested using a stratified logrank test at the two-sided 5% significance level. A stratified Cox proportional regression model will be used to provide point estimates of the HRs with corresponding two-sided 95% confidence intervals. The primary and key secondary endpoints will be tested under a confirmatory hierarchical testing procedure in the order introduced in previous sections, namely the annual eGFR slope, the composite cardiorenal outcome, the composite kidney outcome and the composite cardiovascular outcome.

#### Exploratory endpoints

Analyses of exploratory endpoints include the change from baseline in UACR and the change in health status scores using the EQ-5D-5L.

### Safety

Safety data analyses will be performed using a safety analysis set comprising all randomized patients who take one or more dose of study treatment. Hyperkalaemia is an adverse event of special interest. Prespecified safety analysis assessments include the change in serum potassium from baseline, incidences of serum potassium >5.5 mmol/l and >6.0 mmol/l, hospitalization for hyperkalaemia and permanent discontinuation of study medication due to hyperkalaemia.

### Study oversight

The conduct of the study is being overseen by a steering committee composed of external experts in the areas of nephrology and cardiology. Ongoing safety monitoring during the study is being performed by an external and independent data monitoring committee. FIND-CKD is funded by Bayer AG (the sponsor). The sponsor is responsible for the collection and analysis of data in collaboration with the authors.

## RESULTS

The first patient was enrolled in FIND-CKD in September 2021 and enrolment was completed in May 2023. In total, 3231 patients were screened and 1584 patients were randomized to the study at 283 sites across 24 countries. Most participants failed the screen because they did not meet the eGFR and/or UACR entry criteria (Supplementary data Tables S2 and S3). Of these 1584 patients, 84 were randomized to participate in the decentralized clinical trial (DCT) model. The DCT elements were made available to patients in eight countries in North and Latin America, as well as Asia, Europe and Australia.

Baseline demographics and disease characteristics are summarized in Table 1. A total of 844 (53.3%), 535 (33.8%), 132 (8.3%) and 73 (4.6%) patients were recruited in the regions of Asia, Europe and Oceania, North America and Latin America, respectively. At baseline, the mean age was 54.7 years [standard deviation (SD) 14.3]; 1049 participants (66.2%) in the overall trial population were male and most patients were Asian [*n* = 866 (54.7%)]. Baseline mean eGFR was 46.7 ml/min/1.73 m^2^ (SD 16.1), mean serum potassium was 4.5 mmol/l (SD 0.4) and median UACR was 818.9 mg/g [interquartile range (IQR) 577.4–1244.0]. A total of 844 (53.3%) patients had an eGFR <45 ml/min/1.73 m^2^, 317 (20.0%) had an eGFR ≥60 ml/min/1.73 m^2^ and 592 (37.4%) had a UACR >1000 mg/g.

**Table 1:** Demographics and baseline characteristics (*N* = 1584).

Characteristics	Values
Age (years), mean (SD)	54.7 (14.3)
Male, *n* (%)	1049 (66.2)
Race, *n* (%)	
White	648 (40.9)
Asian	866 (54.7)
Black/African American	37 (2.3)
Other	33 (2.1)
Ethnicity, *n* (%)	
Hispanic	111 (7.0)
Non-Hispanic	1461 (92.2)
Region, *n* (%)	
Europe and Oceania	535 (33.8)
North America	132 (8.3)
Latin America	73 (4.6)
Asia	844 (53.3)
eGFR (ml/min/1.73 m^2^), mean (SD)^a^	46.70 (16.1)
eGFR category (ml/min/1.73 m^2^), *n* (%)	
<25	23 (1.5)
25–<45	821 (51.8)
45–<60	423 (26.7)
≥60	317 (20.0)
UACR (mg/g), median (IQR)	818.90 (577.4–1244.0)
UACR category (mg/g), *n* (%)	
<300	63 (4.0)
300–<1000	929 (58.6)
>1000	592 (37.4)
BMI (kg/m^2^), mean (SD)	27.61 (5.6)
SBP (mmHg), mean (SD)	129.5 (14.1)
DBP (mmHg), mean (SD)	80.0 (9.6)
HbA1c (%), mean (SD)	5.5 (0.4)
Serum potassium (mmol/l), mean (SD)	4.5 (0.4)
Kidney disease aetiology, *n* (%)	
Hypertensive/ischaemic nephropathy	460 (29.0)
Chronic glomerulonephropathies	903 (57.0)
IgAN	417 (26.3)
FSGS	215 (13.6)
Primary FSGS	109 (6.9)
Secondary FSGS	106 (6.7)
Membranous nephropathy	91 (5.7)
Mesangial proliferative glomerulonephritis^b^	17 (1.1)
Other chronic glomerulonephritis	154 (9.7)
Other	57 (3.6)
Unknown	164 (10.4)
History of cardiovascular disease, *n* (%)	
Hypertension	1396 (88.1)
Atherosclerotic cardiovascular disease	189 (11.9)
Myocardial infarction	42 (2.7)
Stroke	82 (5.2)
Peripheral arterial disease	29 (1.8)
Atrial fibrillation	59 (3.7)
Heart failure	35 (2.2)
Concomitant medications, *n* (%)	
RAS inhibitor^c^	1581 (99.8)
ACEIs^c^	435 (27.5)
ARBs^c^	1146 (72.3)
SGLT2 inhibitors	267 (16.9)
Potassium-lowering agents	58 (3.7)
Potassium supplements	20 (1.3)
Beta blockers	403 (25.4)
Diuretics	282 (17.8)
Loop diuretics	128 (8.1)
Thiazide diuretics	116 (7.3)
Calcium channel blockers	794 (50.1)
Statins	851 (53.7)

a Calculated using the CKD-EPI equation.

^b^Defined as the occurrence of one of the following non-prespecified terms: glomerulonephritis membranoproliferative, glomerulonephritis proliferative, mesangioproliferative glomerulonephritis.

^c^According to the protocol, all patients were required to use an ACEI or ARB if tolerated.

BMI: body mass index; DBP: diastolic blood pressure; FSGS: focal segmental glomerulosclerosis; HbA1c: glycated haemoglobin; SBP: systolic blood pressure.

The number of patients taking ACEIs or ARBs at baseline was 1581 (99.8%), as mandated by the protocol. Statins [*n* = 851 (53.7%)] and calcium channel blockers [*n* = 794 (50.1%)] were the most frequently used concomitant medications after RAS inhibitors in the overall population. Diuretics were used by 282 participants (17.8%). In addition, 267 patients (16.9%) were on SGLT2 inhibitors. While patients using SGLT2 inhibitors had a slightly higher baseline median UACR than those who were not receiving SGLT2 inhibitors [871.9 mg/g (IQR 619.2–1409.1) versus 808.3 mg/g (IQR 566.6–1218.5)], baseline characteristics were largely comparable between these subgroups (Table 2). ARBs were prescribed more frequently in Asia compared with the other regions, while ACEIs, diuretics, beta blockers and SGLT2 inhibitors were prescribed less frequently (Supplementary data Table S4).

**Table 2:** Kidney disease characteristics by SGLT2 inhibitor use at baseline.

	Receiving an SGLT2 inhibitor at baseline	
Characteristics	Yes (*n* = 267)	No (*n* = 1317)
eGFR (ml/min/1.73 m^2^), mean (SD)	45.6 (15.7)	46.8 (16.0)
eGFR category (ml/min/1.73 m^2^), *n* (%)		
<25	10 (3.7)	44 (3.3)
25–<45	142 (53.2)	642 (48.7)
45–<60	64 (24.0)	334 (25.4)
≥60	47 (17.6)	279 (21.2)
UACR (mg/g), median (IQR)	871.9 (619.2–1409.1)	808.3 (566.6–1218.5)
UACR category (mg/g), *n* (%)		
<300	6 (2.2)	57 (4.3)
300–≤1000	148 (55.4)	781 (59.3)
>1000	113 (42.3)	479 (36.4)

**Figure 3:**
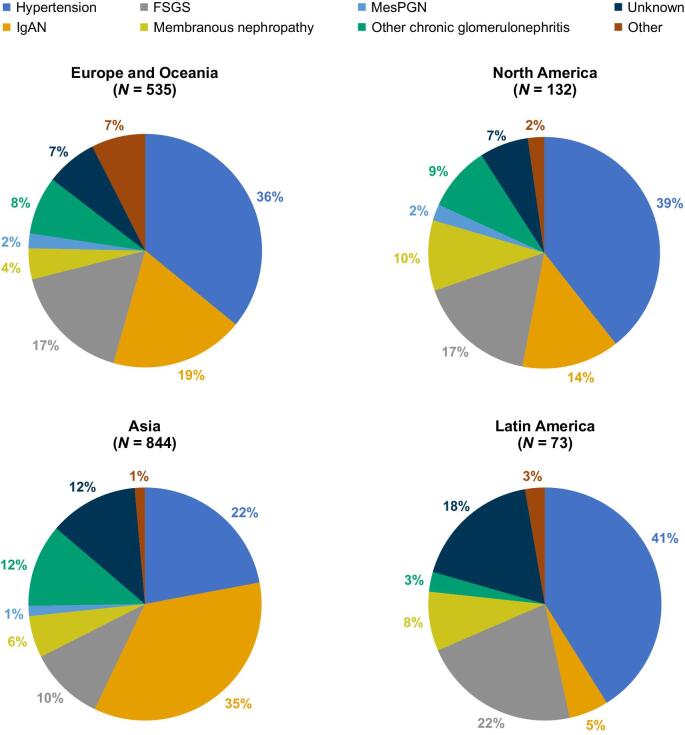
Kidney disease aetiology by region. FSGS: focal segmental glomerulosclerosis; MesPGN: mesangial proliferative glomerulonephritis.

CKD was attributed to hypertensive/ischaemic nephropathy, immunoglobulin A nephropathy (IgAN) and focal segmental glomerulosclerosis in 460 (29.0%), 417 (26.3%) and 215 (13.6%) patients, respectively (Table 1). The aetiology of CKD by region is shown in Fig. 3. Notably, hypertensive/ischaemic nephropathy was the dominant cause of CKD in the regions of Europe and Oceania, Latin America and North America (36–41%), whereas CKD attributed to IgAN was more predominant in Asia (35%). CKD aetiology was confirmed by kidney biopsy in 787 (49.7%) patients at baseline. At baseline, 1396 (88.1%), 189 (11.9%) and 35 (2.2%) patients had hypertension, atherosclerotic cardiovascular disease and heart failure, respectively. Atherosclerotic cardiovascular disease was less prevalent in Asia compared with other regions (Supplementary data Table S4).

## DISCUSSION

The efficacy and safety of the non-steroidal MRA finerenone has been demonstrated in the FIDELIO-DKD and FIGARO-DKD trials [13, 14, 21]. Based on these trials, clinical practice guidelines recommend finerenone as part of the pharmacological treatment to prevent kidney disease progression and cardiovascular events in CKD patients with T2D [22–25]. Both trials excluded patients with CKD without diabetes [13, 14, 21]. The FIND-CKD trial is ongoing to address this knowledge gap and is evaluating the efficacy and safety of finerenone in participants with CKD without diabetes.

Activation of the mineralocorticoid receptor contributes to sodium and water retention, inflammation and fibrosis, which in the long term impairs kidney and cardiovascular function [26]. Small clinical studies in patients with CKD with and without diabetes showed that the steroidal MRAs spironolactone and eplerenone reduced albuminuria and stabilized eGFR decline during 1 year of treatment [27–29]. However, spironolactone and eplerenone increase potassium levels and the risk of hyperkalaemia, particularly in patients with more severe CKD [30, 31]. This side effect has dampened enthusiasm to conduct large clinical trials to determine the long-term effects on clinical kidney outcomes and the lack of sufficient data has limited their use; neither eplerenone nor spironolactone are approved for the treatment of CKD [32, 33]. The pharmacological properties of finerenone are different from the steroidal MRAs [34]. Indeed, in the prespecified pooled analysis of the FIDELIO-DKD and FIGARO-DKD trials (FIDELITY) the incidence of permanent drug discontinuation due to hyperkalaemia was low (1.7%) [21]. In an indirect comparative analysis of FIDELITY and the AMBER trial (NCT03071263), finerenone was associated with a lower risk of hyperkalaemia and discontinuation than spironolactone [35]. Although comparisons across trials should be interpreted carefully, a head-to-head comparison of finerenone and spironolactone in ≈400 patients with heart failure and CKD supports the notion that finerenone is associated with a lower risk of hyperkalaemia than spironolactone [36]. The lower risk of hyperkalaemia with finerenone compared with steroidal MRAs may be attributed to a combination of physicochemical, pharmacokinetic and pharmacodynamic properties, including a short half-life (2–3 hours) and a distinct receptor binding mode with subsequent differential gene expression [12]. The FIND-CKD trial will monitor potassium levels throughout the study and provide a detailed characterization of the effects of finerenone on this safety parameter in patients with CKD due to non-diabetic causes.

The primary endpoint of the FIND-CKD trial is the change in eGFR (eGFR slope) over 32 months of treatment. Emerging evidence supports the GFR slope as a valid surrogate endpoint to establish drug efficacy in clinical trials of CKD progression. A meta-analysis of 66 randomized controlled trials demonstrated a strong association between treatment effects on GFR slope and treatment effects on an established kidney outcome of 57% GFR decline or kidney failure [19]. Importantly, this association was consistent across CKD disease subgroups and the severity of CKD [19]. This analysis also showed that a treatment effect of 0.75 ml/min/1.73 m^2^/year observed in a clinical trial of 1600 participants translates into a reduction in the incidence of 57% GFR decline or kidney failure with very high confidence [19] [median HR 0.74 (95% Bayesian prediction intervals 0.60, 0.89)]. These data also indicated that the association between treatment effects on eGFR slope and the clinical kidney endpoint is strongest for total slope compared with chronic slope, supporting the use of total slope as the primary endpoint, with effects on chronic slope providing supplementary efficacy information. A composite endpoint of ≥57% GFR decline or kidney failure is also used as a key secondary outcome in the FIND-CKD trial, although the trial is not powered to detect statistically significant treatment effects on this outcome [13]. An endpoint based on a larger decline in GFR is being used in FIND-CKD (≥57% versus ≥40% in FIDELIO-DKD), as finerenone may cause an acute, haemodynamically mediated reduction in GFR [13]; this does not reflect the true progression of CKD, potentially confounding the detection of treatment effects. The minimum treatment duration of 32 months was chosen due to post hoc analysis of FIDELIO-DKD based on the total slope difference observed at 32 months [15].

The FIND-CKD trial recruited a broad population with different underlying CKD aetiologies. Table 3 provides further details on kidney characteristics in patients without diabetes in the EMPA-KIDNEY and DAPA-CKD (NCT03036150) studies. Mean age, sex distribution and a history of cardiovascular disease (excluding heart failure) was not different compared with participants without diabetes recruited in the DAPA-CKD or EMPA-KIDNEY trials [37, 38]. The median UACR was higher in FIND-CKD (819 mg/g) than in EMPA-KIDNEY (461 mg/g) but slightly lower than in DAPA-CKD (861 mg/g) [37, 38]. In addition, mean eGFR (ml/min/1.73 m^2^) was highest in FIND-CKD compared with EMPA-KIDNEY and DAPA-CKD (46.7 versus 38.7 and 41.7, respectively) [37, 38].

**Table 3:** Kidney disease characteristics and diagnoses in patients without diabetes by study.

Characteristics	FIND-CKD (*N* = 1584)	DAPA-CKD (*N* = 1398)	EMPA-KIDNEY (*N* = 3570)
eGFR (ml/min/1.73 m^2^), mean (SD)	46.70 (16.1)	41.7 (11.7)	38.7 (15.4)
eGFR category (ml/min/1.73 m^2^), *n* (%)^a^			
<30	241 (15.2)	223 (16.0)	1132 (32.0)
30–<45	603 (38.1)	659 (47.1)	1546 (43.0)
45–<60	423 (26.7)	410 (29.3)	892 (25.0)
≥60	317 (20.0)	106 (7.6)	
UACR (mg/g), median (IQR)	818.9 (577.4–1244.0)	861	461 (128–1117)
UACR category (mg/g), *n* (%)			
<30 (stage A1)	0 (0)	0 (0)	683 (19)
30–300 (stage A2)	63 (4.0)	136 (9.7)	921 (26)
≥300 (stage A3)	1521 (96.0)	1262 (90.3)	1966 (55)
Kidney disease aetiology, *n* (%)			
Hypertensive/ischemic nephropathy	460 (29.0)	494 (35.3)	1044 (29.2)
Any glomerular disease	903 (57.0)	598 (42.8)	1497 (41.9)
IgAN	417 (26.3)	232 (16.6)	758 (21.2)
FSGS	215 (13.6)	93 (6.7)	161 (4.5)
Membranous nephropathy	91 (5.7)	33 (2.4)	83 (2.3)
Glomerulonephritis minimal lesion	7 (0.4)	9 (0.6)	10 (0.3)
Other glomerular disease	154 (9.7)	231 (16.5)	485 (13.6)
Other	57 (3.6)	139 (9.9)	605 (16.9)
Tubulointerstitial disease (including obstructive)	8 (0.5)	117 (8.4)	367 (10.3)
Unknown	164 (10.4)	167 (11.9)	424 (11.9)
Prior kidney biopsy, *n* (%)	787 (49.7)	500 (35.8)	1508 (42.2)

a Calculated by the CKD-EPI equation.

FSGS: focal segmental glomerulosclerosis.

Sources: Wheeler *et al*. [37], EMPA-KIDNEY Collaborative Group [38].

The proportion of patients with glomerulonephritis was higher in FIND-CKD compared with DAPA-CKD and EMPA-KIDNEY and may reflect the larger Asian population recruited in FIND-CKD. Glomerulonephritis is more common in Asia [39]; this was also observed in the FIND-CKD trial, where glomerulonephritis was the most common cause of CKD in Asia. In contrast, in other regions of the world, hypertensive/ischaemic nephropathy was more frequently reported. While hypertensive/ischaemic nephropathy was the dominant cause of CKD in the three studies, the percentage of patients with IgAN was highest in the FIND-CKD study (26.3%) [37, 38]. There were 417 participants with IgAN, a number similar to recently completed phase 3 trials in patients with IgAN [40, 41].

Nearly all participants were using an ACEI or ARB, reflecting the high quality of care of our population. SGLT2 inhibitors are a drug class of particular interest because of their benefits in kidney outcomes. During the recruitment period of our trial, clinical practice guidelines were updated and now recommend SGLT2 inhibitors in addition to an ACEI or ARB to slow CKD progression [4, 42, 43]. In total, 17% of the cohort was using an SGLT2 inhibitor for >4 weeks prior to enrolment in our trial. This number reflects the current ongoing approval and reimbursement process across different regions and dapagliflozin being the only SGLT2 inhibitor with proven kidney benefits in patients with CKD during recruitment. Patients using SGLT2 inhibitors had similar eGFR and UACR levels compared with those not using SGLT2 inhibitors, indicating that they remain at a high risk of progressive kidney function loss and need additional therapies. Previous post hoc analyses from the finerenone phase 3 programme have suggested that finerenone is similarly effective in patients using and not using SGLT2 inhibitors [44, 45]. The FIND-CKD trial will extend these findings to patients without diabetes and CKD. Although these analyses are of interest, they do not answer the question if combined initiation of these two therapies will reduce albuminuria or eGFR decline to a greater extent than monotherapy. The ongoing CONFIDENCE trial (NCT05254002) is designed to prospectively assess the albuminuria-lowering effects of monotherapy with the SGLT2 inhibitor empagliflozin, finerenone and their combination in 807 adults with T2D and CKD [46]. Diuretics were also used in 18% of patients, a percentage that is much lower compared with other recent CKD progression clinical trials [7, 8]. This may also reflect the high proportion of Asian patients where diuretics are less frequently used compared with other regions [47]. In addition, the exclusion of patients with symptomatic heart failure with reduced ejection fraction with a class 1A indication for steroidal MRAs and differences in mean eGFR may both account for the lower use of diuretics in FIND-CKD compared with DAPA-CKD and EMPA-KIDNEY.

A novel aspect of the FIND-CKD trial is that participants are offered the opportunity to participate in the decentralized clinical trial model where all study procedures are performed remotely, i.e. while they are at home. Advances in digital technologies and wearable devices enable remote patient assessment, potentially reducing the need for numerous clinic visits, decreasing the patient’s time commitment, alleviating participant burden and streamlining clinical trial operations [48]. The FIND-CKD trial assesses the feasibility of decentralized clinical trial conduct according to local regulations and legislation in a subset of patients to increase experience and draw lessons for future clinical trials.

In conclusion, FIND-CKD is designed to evaluate the efficacy, safety and tolerability of finerenone compared with placebo in patients with CKD without diabetes. Should a beneficial effect on slowing GFR decline be established, it will expand the role of finerenone for kidney protection beyond T2D.

## Supplementary Material

gfae132_Supplemental_File

## Data Availability

Availability of the data underlying this publication will be determined according to Bayer's commitment to the EFPIA/PhRMA ‘Principles for responsible clinical trial data sharing’. This pertains to scope, time point and process of data access. As such, Bayer commits to sharing, upon request from qualified scientific and medical researchers, patient-level clinical trial data, study-level clinical trial data and protocols from clinical trials in patients for medicines and indications approved in the USA and European Union (EU) as necessary for conducting legitimate research. This applies to data on new medicines and indications that have been approved by the EU and US regulatory agencies on or after 1 January 2014. Interested researchers can use www.vivli.org to request access to anonymized patient-level data and supporting documents from clinical studies to conduct further research that can help advance medical science or improve patient care. Information on the Bayer criteria for listing studies and other relevant information is provided in the member section of the portal. Data access will be granted to anonymized patient-level data, protocols and clinical study reports after approval by an independent scientific review panel. Bayer is not involved in the decisions made by the independent review panel. Bayer will take all necessary measures to ensure that patient privacy is safeguarded.

## FIND-CKD COLLABORATORS

Julio Bittar, Cesar Javier Zaidman, Natalia Cluigt, Miguel Hominal, Paola Aguerre, Fernando Halac, Elizabeth Gelersztein, Mariano Arriola, Rafael Maldonado, Mariano Chahin, David Packham, Darren Lee, Eugenia Pedagogos, Celine Foote, Sunil Badve, Carmel Hawley, Jenny Chen, Nicholas Gray, Marijn Speeckaert, Laura Labriola, Peter Doubel, Bart Maes, Kathleen Claes, Bernard Dubois, Irena Dimitrova, Tsvetelina Vutova, Stefan Ilchev, Svetla Stamova, Yordanka Ivanova, Albena Vasileva, Xiangmei Chen, Shuifu Tang, Xudong Xu, Bicheng Liu, Weiming He, Yani He, Fang Liu, Caili Wang, Lianhua Chen, Jianying Niu, Deguang Wang, Ping Luo, Yuou Xia, Gengru Jiang, Qun Luo, Fang Wang, Menghua Chen, Hongli Lin, Rui Yan, Yinan Li, Qinkai Chen, Junwu Dong, Fei Xiong, Haibo Long, Hong Cheng, Yuehong Li, Juan Du, Fanna Liu, Qingping Chen, Wanhong Lu, Chaosheng Chen, Jianqin Wang, Lei Liu, Min Yang, Gang Long, Yongjun Shi, Wenge Li, Xiangdong Yang, Aicheng Yang, Jianfei Li, Xiaoyan Meng, Martin Prazny, Lucie Hornova, Petr Bucek, Maria Majernikova, Jan Wirth, Jitka Rehorova, Mads Hornum, Jesper Bech, Morten Lindhardt, Ditte Hansen, Line Mortensen, Claus Juhl, Ioannis Boletis, Dorothea Papadopoulou, Evangelos Papachristou, Gerasimos Bamichas, Dimitrios Petras, Chariklia Gouva, Pantelis Sarafidis, Konstantinos Stylianou, Evangelia Ntounousi, Sydney Chi Wai Tang, Cheuk Chun Szeto, Samuel Ka Shun Fung, Sing Leung Lui, Laszlo Kovacs, Aniko Nemeth, Zsolt Zilahi, Tamas Szelestei, Robert Kirschner, Avinash Ignatius, Alan Almeida, Manisha Sahay, Subbiah Arunkumar, Dinesh Khullar, Rajendra Pandey, Sakthirajan Ramanathan, Noble Gracious, Siddharth Mavani, Nomy Levin-Iaina, Benaya Rozen-Zvi, Etty (Esther) Kruzel-Davila, Yosef Haviv, Sydney Ben Chetrit, Pazit Beckerman, Adi Leiba, Gil Chernin, Illia Beberashvili, Orit Kliuk-Ben Bassat, Yael Kenig, Evgeny Farber, Aneliya Parvanova Ilieva, Ciro Esposito, Roberto Minutolo, Gaetano La Manna, Gennaro Santorelli, Maria Cristina Gregorini, Gabriele Donati, Enrico Fiaccadori, Barbara Gidaro, Roberto Cimino, Giuseppe Grandaliano, Izaya Nakaya, Yoshitaka Maeda, Takayuki Toda, Hirokazu Okada, Morimasa Amemiya, Hitoshi Suzuki, Masanori Abe, Hiroshi Nishi, Yoshihiko Kanno, Seiji Ueda, Tetsuro Fujii, Jin Oshikawa, Takehiko Wada, Koichi Tamura, Masahiko Yazawa, Tamio Iwamoto, Tadashi Toyama, Kiyoki Kitagawa, Kohei Uchimura, Yuji Kamijo, Shinji Ako, Kanyu Miyamoto, Taro Misaki, Satoshi Suzuki, Hideaki Shimizu, Yoshiro Fujita, Minamo Ono, Atsushi Yamauchi, Hideki Fujii, Naohiko Fujii, Masaru Matsui, Kengo Kidokoro, Hidetoshi Kanai, Kosuke Masutani, Kiichiro Fujisaki, Yoshirou Nagano, Megumi Nakamura, Mariko Toyoda, Yuichiro Makita, Li Yuan Lee, Chek Loong Loh, Suryati Yakob, Mohd Kamil Ahmad, Kai Quan Lee, Wan Ahmad Hafiz Wan Md Adnan, Muhamad Ali Sk Abdul Kader, Nuzaimin Hadafi Ahmad, Subasni Govindan, Mohamad Zaimi Abdul Wahab, Sadanah Aqashiah Datuk Mazlan, Sergio Irizar Santana, Alfredo Chew Wong, Sandro Avila Pardo, Edmundo Bayram, Rita Birne, Fernando Teixeira e Costa, Joana Silva Costa, Ana Rita Alves, Tiago Pereira, Tatyana Rodionova, Natalia Antropenko, Tatyana Abissova, Elena Zhdanova, Andrey Ezhov, Sufi Muhummad Suhail, Allen Liu, Jimmy Teo, See Cheng Yeo, Ngiap Chuan Tan, SungGyun Kim, Kang Wook Lee, Seok Joon Shin, Byoung-Geun Han, Jangwook Lee, Sang Youb Han, Hye Ryoun Jang, Jung Pyo Lee, Jung Tak Park, Young Sun Kang, So Young Lee, Yong Chul Kim, Sang Ho Lee, Hayne Park, Ji Eun Oh, Yeong Hoon Kim, Bum Soon Choi, Jose Julian Segura de la Morena, Julio Hernandez Jaras, Francisco Martínez Debén, Hanane Bouarich, Pau Llacer Iborra, María José Soler, Jose Gorriz Teruel, Cristina Castro, Josep Cruzado Garrit, Clara Barrios, Yen-Ling Chiu, Hsi-Hsien Chen, Cheng-Chieh Hung, Shuei-Liong Lin, Chien-Te Lee, Ming-Ju Wu, Ping-Fang Chiu, Chiz-Tzung Chang, Hui-Teng Cheng, Kieran McCafferty, Siân Griffin, Priscilla Smith, Timothy Doulton, Thomas Pickett, Arif Khwaja, Radica Alicic, Sreedhara Alla, Sanjiv Anand, Mohamed Atta, Ahmed Awad, Shweta Bansal, Anna Burgner, Alex Chang, Cynthia Christiano, Aditi Gupta, German Hernandez, Aamir Jamal, Eric Kirk, Nelson Kopyt, Wayne Kotzker, Ramon Mendez, Jill Meyer, Ahmadshah Mirkhel, George Newman, Sagar Panse, Pablo Pergola, Mahboob Rahman, Anjay Rastogi, Mark Smith, Jeffrey Turner, Guillermo Umpierrez, Nam Vo, Darren Schmidt, Adam Frome, George Nakhoul, Ronald Ralph, Jonathan Tolins, Jessica Kendrick, Michael Quadrini, Sadaf Elahi, Sergio Trevino Manllo, Wen-Yuan Chiang, Jany Moussa, Tina Thethi.
