# Supplementary material for: Design and baseline characteristics of the Finerenone, in addition to standard of care, on the progression of kidney disease in patients with Non-Diabetic Chronic Kidney Disease (FIND-CKD) randomized trial
Source: Nephrol Dial Transplant. 2024 Jun 11;40(2):308–19. doi: 10.1093/ndt/gfae132 (PMC11852274; doi:10.1093/ndt/gfae132)
Supplement: gfae132_Supplemental_File [file gfae132_Supplemental_File.docx]

# SUPPLEMENTARY MATERIALS

# Plain language summary

Chronic kidney disease is a condition where the kidneys are not working properly. Chronic kidney disease can get worse over time. Eventually, the kidneys may stop working, resulting in the need for a procedure called dialysis which removes waste products from the body. In addition, people with chronic kidney disease are more likely to develop heart disease and tend to have a shorter life expectancy than people without kidney disease. Here, the authors describe the design of a study in people without diabetes but with chronic kidney disease, called FIND-CKD. FIND-CKD is studying whether a drug called finerenone can slow down the progression of kidney disease. The FIND-CKD study will look at whether finerenone will reduce the long-term decline of estimated glomerular filtration rate, an important measure of how well one’s kidneys are working. Long-term decline in estimated glomerular filtration rate is recognized to be a sign of kidney disease progression. Reducing the amount of decline in estimated glomerular filtration rate with finerenone would show that finerenone can slow down kidney disease progression in people with chronic kidney disease.

## Members of the executive committee/steering committee

Chairmen: Hiddo J.L. Heerspink and Vlado Perkovic.

Members: Rajiv Agarwal, George L. Bakris, David Z.I. Cherney, Carolyn S.P. Lam, Brendon L. Neuen, Katherine R. Tuttle, and Christoph Wanner,

## Independent data monitoring committee

Murray Epstein (Chair), Glenn M. Chertow (Chair), Adeera Levin, Johannes Mann, and Tim Friede (Statistician).

## FIND-CKD: National leaders committee

Pantelis Sarafidis (National leaders coordinator), Rafael Maldonado (Argentina), David Packham (Australia), Marijn Speeckaert (Belgium), Svetla Stamova (Bulgaria), Xiangmei Chen (China), Martin Prazny (Czechia), Mads Hornum (Denmark), Evangelos Papachristou (Greece), Sydney Chi Wai Tang (Hong Kong), Laszlo Kovacs (Hungary), Sanjay Agarwal (India), Benaya Rozen-Zvi (Israel), Roberto Minutolo (Italy), Masaomi Nangaku (Japan), Rita Birne (Portugal), Wan Ahmad Hafiz Wan Md Adnan (Malaysia), Alfredo Chew Wong (Mexico), Vladimir Dobronravov (Russia), Yeo See Cheng (Singapore), María José Soler (Spain), Chien-Te Lee (Taiwan), Kieran McCafferty (UK), Pablo Pergola (USA), and Susanne Nicholas (USA).

## Participating countries and investigators: FIND-CKD

Julio Bittar, Centro de Rehabilitacion Cardiovascular | San Luis, Argentina, Argentina; Cesar Javier Zaidman, Centro de Investigacion y Prevencion Cardiovascular | Recoleta Headquarters, Argentina; Natalia Cluigt, Instituto de Investigaciones Clinicas de Mar del Plata | Mar del Plata, Argentina, Argentina; Miguel Hominal, Centro de Investigaciones Clínicas del Litoral | Investigation Department, Argentina; Paola Aguerre, Asociacion de Fomento y Sala de Primeros Auxilios Alberto Mignaburu | Berazategui, Argentina, Argentina; Fernando Halac, Centro de Investigaciones Metabolicas | Ciudad de Autonoma de Buenos Aires, Argentina, Argentina; Elizabeth Gelersztein, CEDIC Centro de Investigación Clínica | Buenos Aires, Argentina, Argentina; Mariano Arriola, Clinica de Nefrologia, Urologia y Enfermedades Cardiovascualares - Santa Fe, Argentina, Argentina; Rafael Maldonado, Clinica Privada Velez Sarsfield | Nephrology and Transplantation Service, Argentina; Mariano Chahin, Centro de Investigaciones Medicas Lanus | Research Department, Argentina; David Packham, Melbourne Renal Research Group, Australia; Darren Lee, Eastern Health Integrated Renal Service, Australia; Eugenia Pedagogos, Sunshine Hospital, Australia; Celine Foote, Concord Repatriation General Hospital, Australia; Sunil Badve, St George Hospital, Australia; Carmel Hawley, Princess Alexandra Hospital Australia, Australia; Jenny Chen, Illawarra Shoalhaven Local Health District, Australia; Nicholas Gray, Sunshine Coast University Hospital, Australia; Marijn Speeckaert, UZ Gent, Belgium; Laura Labriola, Cliniques universitaires Saint-Luc, Belgium; Peter Doubel, AZ Groeninge, Belgium; Bart Maes, AZ Delta, Belgium; Kathleen Claes, UZ Leuven, Belgium; Bernard Dubois, CHU de Liège, Belgium; Irena Dimitrova, Medical Center Hera | Montana Branch, Bulgaria; Tsvetelina Vutova, Diagnostic-Consultative center XX | Bulgaria; Stefan Ilchev, Medical Center ’’Nevromedics’’, Bulgaria; Svetla Stamova, Medical Center ’’4Life’’, Bulgaria; Yordanka Ivanova, Multiprofile Hospital for Active Treatment St. Ivan Rilski - Gorna Oryahovitsa | Nephrology Department, Bulgaria; Albena Vasileva, Viva Phoenix Medical Center | Clinical Trials Department, Bulgaria; Xiangmei Chen, Chinese PLA General Hospital, China; Shuifu Tang, The First Affiliated Hospital of Guangzhou University of TCM, China; Xudong Xu, Central Hospital of Minhang District, Shanghai, China; Bicheng Liu, Zhongda Hospital Southeast University, China; Weiming He, Jiangsu Province Hospital of Chinese Medicine, China; Yani He, Daping Hospital, 3rd Affil Hosp. 3rd Military Med Univ PLA, China; Fang Liu, Sichuan University West China Hospital, China; Caili Wang, The First Affiliated Hospital of Baotou Medical College, China; Lianhua Chen, Huai'an First People's Hospital, Nanjing Medical University, China; Jianying Niu, Shanghai Fifth People's Hospital,Fudan University, China; Deguang Wang, The Second Hospital of Anhui medical university, China; Ping Luo, The Second Hospital of Jilin University, China; Yuou Xia, Siping Central Hospital, China; Gengru Jiang, Xinhua Hos Affiliated to SH Jiaotong Uni School of Medicine, China; Qun Luo, Hwa Mei Hospital, University of Chinese Academy Sciences, China; Fang Wang, SichuanAcademyofMedicalSciences&SichuanProvincialPeople'sHos, China; Menghua Chen, General Hospital of Ningxia Medical University, China; Hongli Lin, The First Affiliated Hospital of Dalian Medical University, China; Rui Yan, The Affiliated Hospital of Guizhou Medical University, China; Yinan Li, The First Affiliated Hospital of Xiamen University, China; Qinkai Chen, The First Affiliated Hospital of NanChang University, China; Junwu Dong, Puai Hospital,Tongji Medical College, Huazhong Univ Sci&Tech, China; Fei Xiong, Wuhan Hospital Of Traditional Chinese And Western Medicine, China; Haibo Long, Zhujiang Hospital of Southern Medical University, China; Hong Cheng, Beijing Anzhen Hospital, Capital Medical University, China; Yuehong Li, Beijing Tsinghua Changgung Hospital, China; Juan Du, Renmin Hosp., Wuhan Univ., China; Fanna Liu, The First Affiliated Hospital of Jinan University, China; Qingping Chen, Jiangxi PingXiang people's Hospital, China; Wanhong Lu, The First Affiliated Hospital of Xi'an Jiaotong University, China; Chaosheng Chen, The First Affiliated Hospital of Wenzhou Medical Univ (New), China; Jianqin Wang, Lanzhou University Second Hospital, China; Lei Liu, The First Affiliated Hospital of Bengbu Medical College, China; Min Yang, 1st Peopl's Hosp of Changzhou 3rd Affil Hosp of Soochow Univ, China; Gang Long, Tianjin Union Medicine Centre (People's Hospital of Tianjin), China; Yongjun Shi, Huizhou Central People's Hospital, China; Wenge Li, China-Japan Friendship Hospital, China; Xiangdong Yang, Qilu Hosp., Shandong Univ., China; Aicheng Yang, Jiangmen Wuyi Hospital of traditional Chinese Medicine, China; Jianfei Li, People's Hospital of Liuzhou City, China; Xiaoyan Meng, Liuzhou Worker's Hospital, China; Martin Prazny, Vseobecna fakultni nemocnice v Praze, Czech Republic; Lucie Hornova, Nefrologicka ambulance, Czech Republic; Petr Bucek, MUDr.Petr Bucek s.r.o., Czech Republic; Maria Majernikova, Nemocnice Znojmo, Czech Republic; Jan Wirth, Privamed s.r.o., Czech Republic; Jitka Rehorova, Fakultní nemocnice Brno - Interni gastroenterologicka klinika, Czech Republic; Mads Hornum, Rigshospitalet, Copenhagen University Hospital | Department of Nefrology, Denmark; Jesper Bech, Region Midtjylland | Regionshospitalet Godstrup - Nephrology Department, Denmark; Morten Lindhardt, Region Sjælland | Holbaek Sygehus - Cardiology department, Denmark; Ditte Hansen, Herlev Hospital | Nephrology Department, Denmark; Line Mortensen, Odense University Hospital | Department of Kidney Medicine, Denmark; Claus Juhl, Hospital of South West Jutland | Department of Endocrinology Research, Denmark; Ioannis Boletis, LAIKO General Hospital of Athens, Greece; Dorothea Papadopoulou, Papageorgiou General Hospital of Thessaloniki, Greece; Evangelos Papachristou, University General Hospital of Patras | Nephrology Clinic, Greece; Gerasimos Bamichas, PAPANIKOLAOU General Hospital Thessaloniki, Greece; Dimitrios Petras, HIPPOKRATION General Hospital of Athens, Greece; Chariklia Gouva, General Hospital of Arta | Nephrology Department, Greece; Pantelis Sarafidis, Hippokration General Hospital of Thessaloniki, Greece; Konstantinos Stylianou, University General Hospital of Heraklion, Greece; Evangelia Ntounousi, Univ. General Hospital of Ioannina, Greece; Sydney Chi Wai Tang, Queen Mary Hospital, Hong Kong; Cheuk Chun Szeto, Prince of Wales Hospital, Hong Kong; Samuel Ka Shun Fung, Princess Margaret Hospital, Hong Kong; Sing Leung Lui, Tung Wah Hospital, Hong Kong; Laszlo Kovacs, Vas Varmegyei Markusovszky Egyetemi Oktatokorhaz, Hungary; Aniko Nemeth, Kanizsai Dorottya Hospital, Hungary; Zsolt Zilahi, Medifarma-98 Egeszsegugyi, Kereskedelmi es Szolgaltato Kft., Hungary; Tamas Szelestei, Somogy Varmegyei Kaposi Mor Oktato Korhaz, Hungary; Robert Kirschner, Kistarcsai Flor Ferenc Korhaz, Hungary; Avinash Ignatius, Noble Hospital, India; Alan Almeida, P. D. Hinduja Hospital and Medical Research Centre, India; Manisha Sahay, Osmania General Hospital, India; Subbiah Arunkumar, All India Institute of Medical Sciences, India; Dinesh Khullar, Max Super Speciality Hospital, Saket, India; Rajendra Pandey, Institute of Post-Graduate Medical Education and Research, India; Sakthirajan Ramanathan, Madras Medical College, India; Noble Gracious, Government Medical College Thiruvananthapuram, India; Siddharth Mavani, Mavani Research Center, India; Nomy Levin-Iaina, Barzilai Medical Center | Department of Nephrology and Hypertension, Israel; Benaya Rozen-Zvi, Clalit Health Services Rabin Medical Center-Beilinson Campus, Israel; Etty (Esther) Kruzel-Davila, Health Corporation of Galilee Medical Center, Israel; Yosef Haviv, Soroka University Medical Center, Israel; Sydney Ben Chetrit, Meir Medical Center, Israel; Pazit Beckerman, Chaim Sheba Medical Center, Israel; Adi Leiba, Assuta Ashdod, Israel; Gil Chernin, Kaplan Medical Center, Israel; Illia Beberashvili, Shamir Medical Center (Assaf Harofeh), Israel; Orit Kliuk-Ben Bassat, Tel-Aviv Sourasky Medical Center, Israel; Yael Kenig, Rambam Health Corporation, Israel; Evgeny Farber, Poria Medical Center | Nephrology and Hypertension Department, Israel; Aneliya Parvanova Ilieva, Istituto Ricerche Farmacologiche Mario Negri IRCCS, Italy; Ciro Esposito, IRCCS Istituti Clinici Scientifici Maugeri SpA SB, Italy; Roberto Minutolo, A.O.U. Luigi Vanvitelli, Italy; Gaetano La Manna, A.O.U. di Bologna Policlinico S.Orsola Malpighi, Italy; Gennaro Santorelli, ASST di Monza, Italy; Maria Cristina Gregorini, AUSL-IRCCS di Reggio Emilia, Italy; Gabriele Donati, A.O.U. di Modena - Policlinico, Italy; Enrico Fiaccadori, A.O.U. di Parma, Italy; Barbara Gidaro, ASST Ovest Milanese, Italy; Roberto Cimino, ASST Rhodense, Italy; Giuseppe Grandaliano, Fondazione Policlinico Universitario Agostino Gemelli IRCCS, Italy; Izaya Nakaya, Iwate Prefectural Central Hospital, Japan; Yoshitaka Maeda, JA Toride Medical Center, Japan; Takayuki Toda, Tsuchiura Kyodo General Hospital, Japan; Hirokazu Okada, Saitama Medical University Hospital, Japan; Morimasa Amemiya, Japanese Red Cross Saitama Hospital, Japan; Hitoshi Suzuki, Juntendo University Urayasu Hospital, Japan; Masanori Abe, Nihon University Itabashi Hospital, Japan; Hiroshi Nishi, The University of Tokyo Hospital, Japan; Yoshihiko Kanno, Tokyo Medical University Hospital, Japan; Seiji Ueda, Juntendo University Hospital, Japan; Tetsuro Fujii, Hiratsuka Kyosai Hospital, Japan; Jin Oshikawa, Yokohama Sakae Kyosai Hospital, Japan; Masahiro Koizumi, Tokai University Hospital, Japan; Koichi Tamura, Yokohama City University Hospital, Japan; Takehiko Wada, Tokai University Hospital, Japan; Koichi Tamura, Yokohama City University Hospital, Japan; Masahiko Yazawa, St. Marianna University Hospital, Japan; Tamio Iwamoto, Saiseikai Yokohamashi Nanbu Hospital, Japan; Tadashi Toyama, Kanazawa University Hospital, Japan; Kiyoki Kitagawa, National Hospital Organization Kanazawa Medical Center, Japan; Kohei Uchimura, University of Yamanashi Hospital, Japan; Yuji Kamijo, Shinshu University Hospital, Japan; Shinji Ako, Matsumoto City Hospital, Japan; Kanyu Miyamoto, Central Japan International Medical Center, Japan; Taro Misaki, Seirei Hamamatsu General Hospital, Japan; Satoshi Suzuki, Kainan Hospital, Japan; Hideaki Shimizu, Daido Clinic, Japan; Yoshiro Fujita, Chubu Rosai Hospital, Japan; Minamo Ono, Nagoya City University Hospital, Japan; Atsushi Yamauchi, Osaka Rosai Hospital, Japan; Hideki Fujii, Kobe University Hospital, Japan; Naohiko Fujii, Hyogo Prefectural Nishinomiya Hospital, Japan; Masaru Matsui, Nara Prefecture General Medical Center, Japan; Kengo Kidokoro, Kawasaki Medical School Hospital, Japan; Hidetoshi Kanai, Kokura Memorial Hospital, Japan; Kosuke Masutani, Fukuoka University Hospital, Japan; Kiichiro Fujisaki, Aso Iizuka Hospital, Japan; Masao Ishii, Social Medical Corporation the Chiyukai foundation Fukuoka Wajiro Hospital, Japan; Yoshirou Nagano, Social Medical Corporation the Chiyukai foundation Fukuoka Wajiro Hospital, Japan; Megumi Nakamura, Saga-Ken Medical Centre Koseikan, Japan; Mariko Toyoda, Japanese Red Cross Kumamoto Hospital, Japan; Yuichiro Makita, Koshigaya Municipal Hospital, Japan; Li Yuan Lee, Hospital Seri Manjung, Malaysia; Chek Loong Loh, Hospital Raja Permaisuri Bainun, Malaysia; Suryati Yakob, Hospital Selayang, Malaysia; Mohd Kamil Ahmad, Hospital Tengku Ampuan Afzan, Malaysia; Kai Quan Lee, Hospital Sultan Abdul Halim, Malaysia; Wan Ahmad Hafiz Wan Md Adnan, University Malaya Medical Centre, Malaysia; Muhamad Ali Sk Abdul Kader, Hospital Pulau Pinang, Malaysia; Nuzaimin Hadafi Ahmad, Hospital Pakar Sultanah Fatimah, Malaysia; Subasni Govindan, Hospital Tuanku Jaafar | Nephrology Department, Malaysia; Mohamad Zaimi Abdul Wahab, Hospital Kuala Lumpur, Malaysia; Sadanah Aqashiah Datuk Mazlan, Hospital Kajang, Malaysia; Sergio Irizar Santana, SINACOR | Culiacan, Mexico, Mexico; Alfredo Chew Wong, Sitio Alfredo Chew Wong | Aguascalientes, Mexico, Mexico; Sandro Avila Pardo, Sociedad de Metabolismo y Corazon S.C. | Veracruz, Mexico, Mexico; Edmundo Bayram, Fundacion Cardiovascular de Aguascalientes | Aguascalientes, Mexico, Mexico; Rita Birne, Centro Hospitalar de Lisboa Ocidental | Nephrology Department, Portugal; Fernando Teixeira e Costa, Hospital Garcia de Orta | Nephrology Department, Portugal; Joana Silva Costa, Centro Hospitalar de Leiria | Santo Andre Hospital - Nephrology Department, Portugal; Ana Rita Alves, Centro Hospitalar do Medio Tejo | Unidade de Torres Novas - Nephrology Department, Portugal; Tiago Pereira, Centro Hospitalar Universitario de Lisboa Central | Hospital Curry Cabral - Nephrology Department, Portugal; Tatyana Rodionova, Saratov City Clinical Hospital #9, Russia; Natalia Antropenko, City Clinical Hospital of Emergency Care #1, Russia; Tatyana Abissova, Regional Clinical Hospital Yaroslavl, Russia; Elena Zhdanova, Voronezh Regional Clinical Consultancy-Diagnostic Center, Russia; Andrey Ezhov, Izhevsk City Clinical Hospital #9, Russia; Sufi Muhummad Suhail, Singapore General Hospital, Singapore; Allen Liu, Khoo Teck Puat Hospital, Singapore; Jimmy Teo, National University Hospital, Singapore; See Cheng Yeo, Tan Tock Seng Hospital, Singapore; Ngiap Chuan Tan, SingHealth Polyclinics - Pasir Ris, Singapore; SungGyun Kim, Hallym University Sacred Heart Hospital, South Korea; Kang Wook Lee, Chung Nam National University Hospital, South Korea; Seok Joon Shin, The Catholic University of Korea, Incheon St.Mary's Hospital, South Korea; Byoung-Geun Han, Yonsei University Wonju Christian Hospital, South Korea; Jangwook Lee, Dongguk University Ilsan Hospital, South Korea; Sang Youb Han, Inje University Ilsan Paik Hospital, South Korea; Hye Ryoun Jang, Samsung Medical Center, South Korea; Jung Pyo Lee, Seoul National University Boramae Medical Center, South Korea; Jung Tak Park, Severance Hospital, Yonsei University Health System, South Korea; Young Sun Kang, Korea University Ansan Hospital, South Korea; So Young Lee, BUNDANG CHA HOSPITAL, South Korea; Yong Chul Kim, Seoul National University Hospital, South Korea; Sang Ho Lee, Kyung Hee University Hospital at Gangdong, South Korea; Hayne Park, Kangnam Sacred Heart Hospital, South Korea; Ji Eun Oh, Kangdong Sacred Heart Hospital, South Korea; Yeong Hoon Kim, Inje University Busan Paik Hospital, South Korea; Bum Soon Choi, The Catholic Univ. of Korea Eunpyeong St. Mary's Hospital, South Korea; Jose Julian Segura de la Morena, Hospital Universitario 12 de Octubre | Department of Nephrology, Spain; Julio Hernandez Jaras, La Fe University and Polytechnic Hospital | Nephrology Department, Spain; Francisco Martínez Debén, Complexo HU Ferrol | Nefrología, Spain; Hanane Bouarich, Hospital Principe de Asturias, Spain; Pau Llacer Iborra, Hospital Universitario Ramon y Cajal | Departamento de Medicina Interna, Spain; María Soler Romero, Ciutat Sanitaria i Universitaria de la Vall d'Hebron, Spain; Jose Gorriz Teruel, Hospital Clinico Universitario de Valencia | Nephrology Department, Spain; Cristina Castro, Hospital Universitario Dr. Peset, Spain; Josep Cruzado Garrit, Hospital Universitari de Bellvitge | Bellvitge Biomedical Research Institute - Nephrology Department, Spain; Clara Barrios, Hospital del Mar | Nephrology Department, Spain; Yen-Ling Chiu, Far Eastern Memorial Hospital | Nephrology Department, Taiwan; Hsi-Hsien Chen, Taipei Medical University | Clinical Research Center, Taiwan; Cheng-Chieh Hung, Chang Gung Memorial Hospital at Linkou, Taiwan; Shuei-Liong Lin, National Taiwan University Hospital, Taiwan; Chien-Te Lee, Chang Gung Memorial Hospital Kaohsiung, Taiwan; Ming-Ju Wu, Taichung Veterans General Hospital, Taiwan; Ping-Fang Chiu, Changhua Christian Hospital, Taiwan; Chiz-Tzung Chang, China Medical University Hospital, Taiwan; Hui-Teng Cheng, National Taiwan University Hospital, Hsin-Chu Branch, Taiwan; Kieran McCafferty, Royal London Hospital, United Kingdom; Siân Griffin, University Hospital of Wales, United Kingdom; Priscilla Smith, King's College Hospital, United Kingdom; Timothy Doulton, Kent and Canterbury Hospital, United Kingdom; Thomas Pickett, Gloucestershire Royal Hospital, United Kingdom; Arif Khwaja, Northern General Hospital, United Kingdom; Radica Alicic, Providence Medical Research Center, United States; Sreedhara Alla, Northwest Louisiana Nephrology, United States; Sanjiv Anand, Utah Kidney Research Institute | Salt Lake City, UT, United States; Mohamed Atta, Johns Hopkins University School of Medicine | Division of Nephrology, United States; Ahmed Awad, Clinical Research Consultants, United States; Shweta Bansal, University of Texas Health Science Center at San Antonio | Department of Medicine - Division of Nephrology, United States; Anna Burgner, Vanderbilt University Medical Center, United States; Alex Chang, Geisinger Medical Center, United States; Cynthia Christiano, East Carolina University | Nephrology & Hypertension, United States; Aditi Gupta, University of Kansas, United States; German Hernandez, El Paso Medical Research Institute (MedResearch Inc) | El Paso, TX, United States; Aamir Jamal, North America Research Institute | NARI San Dimas, CA - Dr. Jamal, United States; Eric Kirk, Eastern Nephrology Associates | Wilmington, United States; Nelson Kopyt, Northeast Clinical Research Center | Bethlehem, PA, United States; Wayne Kotzker, Florida Kidney Physicians - Boca Raton, United States; Ramon Mendez, Mendez Center For Clinical Research | Woodbridge, VA, United States; Jill Meyer, California Institute of Renal Research - Chula Vista, United States; Ahmadshah Mirkhel, Nephrology Associates of Northern Virginia | Fairfax, VA, United States; George Newman, Knoxville Kidney Center, United States; Sagar Panse, Central Georgia Kidney Specialist, United States; Pablo Pergola, Clinical Advancement Center, PLLC, United States; Mahboob Rahman, University Hospitals | UH Cleveland Medical Center - Division of Nephrology & Hypertension Research, United States; Anjay Rastogi, University of California, Los Angeles | UCLA Health Division of Nephrology, United States; Mark Smith, Nephrology Associates, PC | Southeastern Clinical Research Institute, LLC., United States; Jeffrey Turner, Yale University | Nephrology Clinical Research, United States; Guillermo Umpierrez, Emory University | Endocrinology Research, United States; Nam Vo, Mountain Kidney & Hypertension Associates, United States; Darren Schmidt, University of New Mexico, United States; Adam Frome, DaVita Clinical Research | North Houston, TX, United States; George Nakhoul, Cleveland Clinic | Urological and Kidney Department, United States; Ronald Ralph, DaVita Clinical Research | Houston, TX, United States; Jonathan Tolins, DaVita Clinical Research | Edina, MN, United States; Jessica Kendrick, University of Colorado | Renal Research Office, United States; Michael Quadrini, Nephrology Consultants, LLC, United States; Sadaf Elahi, NANI Research, LLC, United States; Sergio Trevino Manllo, Gamma Medical Research, Inc., United States; Wen-Yuan Chiang, United Clinical Research & Innovations, United States; Jany Moussa, Wichita Nephrology Group, PA, United States; Tina Thethi, Advent Health, United States.

Table S1. Eligibility criteria

| **Inclusion criteria** |
| --- |
| 1. ≥18 years of age inclusive at the time of signing the informed consent 2. A clinical diagnosis of chronic kidney disease and:  - eGFR ≥25 but <60 mL/min/1.73 m^2^ and UACR of ≥200 but <500 mg/g at screening,^a^ or eGFR ≥25 but <90 mL/min/1.73 m^2^ and UACR of ≥500 but ≤3500 mg/g at screening, and - Documentation of albuminuria/proteinuria^b^ in the patient’s medical records at least 3 months prior to screening. If no value is available prior to pre-screening, the pre-screening can be used to identify patients  1. Stable and maximum tolerated labelled dose of an ACEI or ARB for at least 4 weeks prior to screening as documented in the patient’s medical records 2. Serum potassium ≤4.8 mmol/L at screening 3. Male and/or female; women of childbearing potential can only be included in the study if a pregnancy test is negative at the screening visit and if they agree to use adequate contraception during the study 4. Capable of giving signed informed consent |
| **Exclusion criteria** |
| 1. Established diagnosis of type 1 or 2 diabetes mellitus, or HbA1c ≥6.5% (48 mmol/mol) 2. Autosomal dominant or autosomal recessive polycystic kidney disease 3. Lupus nephritis or ANCA-associated vasculitis or any other primary or secondary kidney disease requiring immunosuppressive therapy within 6 months prior to screening 4. History of organ transplantation 5. Acute kidney injury requiring dialysis within 6 months prior to screening 6. Uncontrolled arterial hypertension with mean sitting systolic blood pressure ≥160 mmHg, mean sitting diastolic blood pressure ≥100 mmHg at screening 7. Symptomatic heart failure with reduced ejection fraction with class 1A indication for MRAs 8. UACR >3500 mg/g at the screening visit^c^ 9. Cardiovascular event within 3 months prior to screening (heart failure decompensation, myocardial infarction, stroke, transient ischemic attack, pulmonary embolism, elective coronary artery bypass grafting) or elective percutaneous coronary intervention within 1 month prior to screening 10. Known hypersensitivity to the study treatment (active treatment or excipients) 11. Addison’s disease 12. Hepatic insufficiency classified as Child-Pugh C 13. Concomitant therapy with eplerenone, spironolactone, esaxerenone, any renin inhibitor, sacubitril/valsartan combination, or potassium-sparing diuretic which cannot be discontinued at least 4 weeks prior to the screening visit 14. Concomitant therapy with both ACEI and ARBs in case one of those cannot be discontinued at least 4 weeks prior to the screening visit 15. Concomitant therapy with potent CYP3A4 inhibitors or moderate and potent inducers (to be stopped at least 7 days before randomization) |

^a^Capped at approximately 10% of the total population. ^b^Quantitative or semiquantitative measurement documented in the medical records. ^c^One re-assessment is allowed in case UACR is >3500 mg/g in one of the three urine samples collected at the screening visit.
ACEI, angiotensin converting enzyme inhibitor; ANCA, antineutrophil cytoplasmic antibody; ARB, angiotensin receptor blocker; CYP3A4, cytochrome P450 3A4; eGFR, estimated glomerular filtration rate; HbA1c, glycosylated haemoglobin; MRA, mineralocorticoid receptor antagonist; UACR, urinary albumin/creatinine ratio.

Table S2. Disposition: End of screening (all enrolled participants)

| **Disposition** | ***N =* 3231 (100%)** |
| --- | --- |
| Completed screening | 1584 (49.0%) |
| Did not complete screening | 1647 (51.0%) |
| Primary reason |  |
| COVID-19 pandemic related: other | 2 (<0.1%) |
| Logistical reason | 3 (<0.1%) |
| Met eligibility criteria but not needed | 60 (1.9%) |
| Other | 22 (0.7%) |
| Physician decision | 4 (0.1%) |
| Screen failure | 1477 (45.7%) |
| Sponsor request | 6 (0.2%) |
| Participant decision | 73 (2.3%) |

COVID-19, coronavirus disease 2019.

Table S3. Participants with inclusion criteria not met/exclusion criteria met (all screening failures)

| **Did not complete screening** | ***N* = 1647 (100%)^a^** |
| --- | --- |
| **Failed eligibility criteria** | **1493 (90.6%)^b^** |
| **Any inclusion criterion not met** | **1349 (81.9%)** |
| A clinical diagnosis of chronic kidney disease and eGFR ≥25 to <60 mL/min/1.73 m^2^ and UACR of ≥200 to  <500 mg/g at screening or eGFR ≥25 to <90 mL/min/1.73 m^2^ and UACR of ≥500 to ≤3500 mg/g at screening, and documentation of albuminuria/proteinuria in the participant’s medical records at least 3 months prior to screening.  If no value is available prior to pre-screening, the pre-screening could be used to identify participants | 1147 (69.6%) |
| K+ ≤4.8 mmol/L at screening | 197 (12.0%) |
| Capable of giving signed informed consent | 59 (3.6%) |
| Stable and maximum tolerated labelled dose of an ACEI or ARB for at least 4 weeks prior to screening as documented in the participant’s medical records | 31 (1.9%) |
| Participant must be ≥18 years of age, at the time of signing the informed consent | 14 (0.9%) |
| Participants of childbearing potential can only be included in the study if a pregnancy test is negative at the Screening Visit and if they agree to use adequate contraception. Contraceptive use by women should be consistent with local regulations regarding the methods of contraception for those participating in clinical studies. Women are considered not of childbearing potential if they fulfil the criteria specified in the protocol | 4 (0.2%) |
| **Any exclusion criterion met** | **261 (15.8%)** |
| Established diagnosis of type 1 or 2 diabetes mellitus, or HbA1c ≥6.5% (4.8 mmol/mol) | 102 (6.2%) |
| UACR >3500 mg/g at the screening visit. (Note: one re-assessment is allowed in case UACR is >3500 mg/g in one of the three urine samples collected at the screening visit) | 68 (4.1%) |
| Uncontrolled arterial hypertension with mean sitting systolic blood pressure ≥160 mmHg and mean sitting diastolic blood pressure ≥100 mmHg at screening | 61 (3.7%) |
| Any other history, condition, therapy, or uncontrolled intercurrent illness that could, in the opinion of the investigator, affect compliance with study requirements | 12 (0.7%) |
| Lupus nephritis or ANCA-associated vasculitis or any other primary or secondary kidney disease requiring immunosuppressive therapy within 6 months prior to screening | 9 (0.5%) |
| Concomitant therapy with eplerenone, spironolactone, esaxerenone, any renin inhibitor, sacubitril/valsartan combination, or potassium-sparing diuretic that cannot be discontinued at least 4 weeks prior to the Screening Visit | 9 (0.5%) |
| Any other condition or therapy that will make the participant unsuitable for the study and will not allow participation for the full planned study period (e.g. active malignancy or other condition limiting life expectancy to less than 12 months) | 6 (0.4%) |
| Pregnant or breast-feeding or intention to become pregnant during the study | 5 (0.3%) |
| Concomitant therapy with both ACEIs and ARBs in case one of those cannot be discontinued at least 4 weeks prior to the Screening Visit | 4 (0.2%) |
| Concomitant therapy with potent CYP3A4 inhibitors or moderate and potent inducers (to be stopped at least 7 days before randomization) | 4 (0.2%) |
| Autosomal dominant or autosomal recessive polycystic kidney disease | 3 (0.2%) |
| CV event within 3 months prior to screening (heart failure decompensation, myocardial infarction, stroke, transient ischemic attack, pulmonary embolism, elective coronary intervention within 1 months prior to screening) | 3 (0.2%) |
| Concomitant therapy with potent CYP3A4 inhibitors or inducers and/or the moderate CYP3A4 inhibitor erythromycin (to be stopped at least 7 days before randomization) | 3 (0.2%) |
| Simultaneous participation in another interventional clinical study (e.g. Phase 1 to 3 clinical studies) or treatment with another investigational medicinal product within 30 days prior to randomization | 3 (0.2%) |
| History of organ transplantation | 2 (0.1%) |
| Acute kidney injury requiring dialysis within 6 months prior to screening | 2 (0.1%) |
| Symptomatic heart failure with reduced ejection fraction with class 1A indication for MRAs | 2 (0.1%) |
| Known hypersensitivity to the study treatment (active substance or excipients) | 2 (0.1%) |
| Addison’s disease | 2 (0.1%) |
| Hepatic insufficiency classified as Child–Pugh C | 2 (0.1%) |
| Previous assignment to treatment during the study | 2 (0.1%) |
| Close affiliation with the investigational site, e.g. a close relative of the investigator or dependent person (e.g. employee or student of the investigational site) | 2 (0.1%) |

^a^A participant could fail more than one eligibility criterion. In total, 170 patients (10.3%) were not randomized for another primary reason (e.g., COVID-19, logistical reason, physician or participant decision, sponsor request, the 10% cap of participants with eGFR of ≥25 to 60 mL/min/1.73 m^2^ and UACR ≥200 to <500 mg/g, or other reasons) rather than as a result of not meeting-eligibility-criteria. ^b^Sixteen patients had a primary reason for not completing screening that was unrelated to eligibility criteria (e.g. hesitant to change standard of care, desire to become pregnant, expected non-compliance).
ACEI, angiotensin converting enzyme inhibitor; ANCA, antineutrophilic cytoplasmic antibody; ARB, angiotensin receptor blocker; COVID-19, coronavirus 2019; CYP3A4, cytochrome P450 enzyme; CV, cardiovascular; eGFR, estimated glomerular filtration rate; HbA1c, glycated haemoglobin; K+, serum potassium; MRA, mineralocorticoid receptor antagonists; UACR, urinary albumin/creatinine ratio.

Table S4. Concomitant medications and atherosclerotic CVD history by region

|  | **Overall (*N* = 1584)** | **Europe and Oceania (*N* = 535)** | **North America (*N* = 132)** | **Asia (*N* = 844)** | **Lain America (*N* = 73)** |
| --- | --- | --- | --- | --- | --- |
| **Concomitant medications,** *n* (%) |  |  |  |  |  |
| RAS inhibitors^a^ | 1581 (99.8) | 534 (99.8) | 131 (99.2) | 843 (99.9) | 73 (100.0) |
| ACEIs^a^ | 435 (27.5) | 243 (45.4) | 44 (33.3) | 125 (14.8) | 23 (31.5) |
| ARBs^a^ | 1146 (72.3) | 291 (54.4) | 87 (65.9) | 718 (85.1) | 50 (68.5) |
| SGLT2 inhibitors | 267 (16.9) | 122 (22.8) | 28 (21.2) | 108 (12.8) | 9 (12.3) |
| Potassium-lowering agents | 58 (3.7) | 19 (3.6) | 5 (3.8) | 32 (3.8) | 2 (2.7) |
| Potassium supplements | 20 (1.3) | 13 (2.4) | 4 (3.0) | 2 (0.2) | 1 (1.4) |
| Beta blockers | 403 (25.4) | 181 (33.8) | 44 (33.3) | 155 (18.4) | 23 (31.5) |
| Diuretics | 282 (17.8) | 152 (28.4) | 48 (36.4) | 67 (7.9) | 15 (20.5) |
| Statins | 851 (53.7) | 315 (58.9) | 74 (56.1) | 428 (50.7) | 34 (46.6) |
| **Atherosclerotic CVD history,** *n* (%) | 189 (11.9) | 76 (14.2) | 21 (15.9) | 79 (9.4) | 13 (17.8) |

^a^According to the protocol, all patients were required to use an ACEI or ARB if tolerated.
ACEI, angiotensin converting enzyme inhibitor; ARB, angiotensin receptor blocker; CVD, cardiovascular disease; RAS, renin–angiotensin system; SGLT2, sodium-glucose cotransporter-2.
